# Electrochemical Impedance Spectroscopy-Based Microfluidic Biosensor Using Cell-Imprinted Polymers for Bacteria Detection

**DOI:** 10.3390/bios14090445

**Published:** 2024-09-18

**Authors:** Shiva Akhtarian, Satinder Kaur Brar, Pouya Rezai

**Affiliations:** 1 Department of Mechanical Engineering, York University, Toronto, ON M3J 1P3, Canada; shivaakh@yorku.com; 2 Department of Civil Engineering, York University, Toronto, ON M3J 1P3, Canada; satinder.brar@lassonde.yorku.ca

**Keywords:** biosensor, bacteria detection, cell-imprinted polymer (CIP), microfluidic, electrochemical sensor

## Abstract

The rapid and sensitive detection of bacterial contaminants using low-cost and portable point-of-need (PoN) biosensors has gained significant interest in water quality monitoring. Cell-imprinted polymers (CIPs) are emerging as effective and inexpensive materials for bacterial detection as they provide specific binding sites designed to capture whole bacterial cells, especially when integrated into PoN microfluidic devices. However, improving the sensitivity and detection limits of these sensors remains challenging. In this study, we integrated CIP-functionalized stainless steel microwires (CIP-MWs) into a microfluidic device for the impedimetric detection of *E. coli* bacteria. The sensor featured two parallel microchannels with three-electrode configurations that allowed simultaneous control and electrochemical impedance spectroscopy (EIS) measurements. A CIP-MW and a non-imprinted polymer (NIP)-MW suspended perpendicular to the microchannels served as the working electrodes in the test and control channels, respectively. Electrochemical spectra were fitted with equivalent electrical circuits, and the charge transfer resistances of both cells were measured before and after incubation with target bacteria. The charge transfer resistance of the CIP-MWs after 30 min of incubation with bacteria was increased. By normalizing the change in charge transfer resistance and analyzing the dose–response curve for bacterial concentrations ranging from 0 to 10^7^ CFU/mL, we determined the limits of detection and quantification as 2 × 10^2^ CFU/mL and 1.4 × 10^4^ CFU/mL, respectively. The sensor demonstrated a dynamic range of 10^2^ to 10^7^ CFU/mL, where bacterial counts were statistically distinguishable. The proposed sensor offers a sensitive, cost-effective, durable, and rapid solution for on-site identification of waterborne pathogens.

## 1. Introduction

The increasing demand for rapid, sensitive, inexpensive, and portable technologies for monitoring pathogenic and indicator bacteria across environmental and medical applications has driven the exploration of innovative detection methods [1,2]. Despite the effectiveness of traditional laboratory-based approaches, such as immunological assays, molecular tests, and cell culturing, these methods are hindered by long analysis times, high costs, and the need for skilled personnel and specialized equipment [3]. In response, point-of-need (PoN) biosensors have emerged as promising solutions, enabling economical, real-time, and on-site detection of bacterial contaminants [4]. PoN biosensors typically consist of two main components: a bio-recognition element and a physical transducer. The bio-recognition element selectively binds to the target analyte, determining the biosensor’s specificity, while the transducer converts the recognition event into a measurable signal. Various transduction techniques, including optical [5,6,7,8,9], mass-based [10], and electrochemical methods [11], have been used in biosensors. Among these, electrochemical detection is particularly advantageous due to its high sensitivity, fast measurements, low cost, and ease of miniaturization, which make it suitable for on-site applications [12,13].

Many bacteria electrochemical biosensors have been developed using bio-recognition materials such as bacteriophages, aptamers, and antibodies [14,15]. While these biosensors show excellent detection performance in laboratory conditions, their real-world deployment is limited due to their high cost and the instability of bioreceptors when exposed to environmental changes, particularly in extreme temperature and humidity conditions.

Cell-imprinted polymers (CIPs) offer a promising alternative, with competitive affinity to target cells similar to biological recognition materials but with lower costs and greater stability across various environmental conditions [16,17]. CIPs are synthesized through molecular imprinting, where a target cell serves as the “template” during fabrication. The template retains its physical and chemical properties in the resulting cavities after polymerization and subsequent elution from the CIP, allowing selective binding in complex biological and environmental samples [17].

In previous works, whole bacteria cells were used to create CIP-coated microwires (CIP-MWs) [18], which showed strong binding efficiency to *E. coli*. These CIP-MWs were integrated into microfluidic devices, creating a miniaturized, cost-effective sensor based on electrical resistance, requiring minimal sample volumes and providing quick analysis [19]. However, the sensor’s limits of detection (LOD) and quantification (LOQ) were relatively high, at 2.1 × 10^5^ CFU/mL and 7.3 × 10^5^ CFU/mL, respectively, along with low sensitivity within a narrow dynamic range of 10^4^ to 10^7^ CFU/mL. These factors require significant improvement to meet performance benchmarks for bacterial detection in practical applications.

In this study, we present the incorporation of CIP-MWs into an affordable electrochemical microfluidic device with a novel design that converts the interaction between CIP cavities and target *E. coli* bacteria into a measurable electrical readout. The impedimetric sensing technique based on EIS measurements is an effective and reliable method for investigating antibody–antigen interactions on electrode surfaces [20,21]. This powerful electrochemical method is capable of detecting subtle changes occurring at the solution–electrode interface, making it suitable for enhancing the performance of CIP-MW-based biosensors. It has been widely employed for characterizing materials, surface modification procedures, and monitoring analyte binding to receptors [22]. Furthermore, the compact nature of the equipment required for EIS facilitates its miniaturization, making it readily amenable to PoN biosensors. Additionally, impedance provides a rapid response, low detection limit, cost-effectiveness, and the ability to conduct real-time sample monitoring. By monitoring the charge transfer resistance of CIP-MWs in a microchannel, we studied the effect of captured bacteria by the CIP-MWs. The developed sensing platform demonstrates enhanced performance with a lower detection limit compared with conductometric detection and holds promise for future integration into handheld devices, enabling sensitive, on-site, and affordable pathogen monitoring.

## 2. Materials and Methods

### 2.1. Materials

Stainless steel microwires (SS-MWs, 125.3 ± 0.5 µm dia., Type 304, Product No. 40944BZ) and silver microwires (Ag-MW, 127 µm dia., Product No. 00303G9) were purchased from Thermo Fisher Scientific™, Haverhill, MA, USA. All chemical reagents, including Luria Broth (LB), methacrylic acid (MAA), acrylamide (AAM), N-vinylpyrrolidone (VP), methylmethacrylate (MMA), ethylene glycol dimethacrylate (EGDMA), 2, 2′-azoisobutyronitrile (AIBN), tetraethyl orthosilicate (TEOS), acetic acid, sulfuric acid, acetonitrile, methanol, potassium ferrocyanide (K_4_[Fe(CN)_6_]), potassium ferricyanide (K_3_[Fe(CN)_6_]), potassium chloride (KCl), and Ag/AgCl paste (60/40), were obtained from Sigma-Aldrich (St. Louis, MO, USA). The *E. coli* OP50 bacteria strain was sourced from the Caenorhabditis Genetics Center (University of Minnesota, Minneapolis, MN, USA).

### 2.2. Bacteria Culturing and Sample Preparation

Bacteria were cultured overnight in LB liquid growth medium within a shaker incubator set at 150 rpm and 37 °C. Bacterial counts were determined using plate culturing and colony counting techniques. For sensor dose–response studies, the cultured bacteria were serially diluted to achieve lower bacterial counts.

### 2.3. Surface Functionalization of MWs

SS-MWs (4 cm long) were cleaned using acetone, followed by ultrasonication for 5 min, and subsequently washed in methanol and DI water. After air-drying, surface oxidation was performed in a 2M sulfuric acid solution for 2 h, followed by rinsing with DI water. Silanization of the hydroxylated SS-MWs’ surfaces was carried out by immersing them in a TEOS-water-methanol solution (2:1:8 vol%) for 30 min. A baking process at 150 °C for 2 h following silanization ensured the formation of a robust silane layer. The SS-MWs were subsequently rinsed three times with ethanol and allowed to air-dry.

Ag-MWs (4 cm long) were cleaned by rinsing with DI water and then with ethanol to remove any contaminants. After air-drying, dip coating was performed by dipping one end of the Ag wire into the Ag/AgCl paste, followed by drying at 150 °C for 30 min.

### 2.4. Fabrication of CIP-MWs

The CIP pre-polymer was previously optimized [18], comprising MMA (5.2 µL), VP (4.2 µL), AAM (21 mg), MAA (180 µL), EGDMA (570 µL), and AIBN (30 mg) dissolved in acetonitrile (2.2 mL), yielding a uniform CIP coating thickness on MWs. AAM provided a hydrophilic backbone, enhancing the solubility and bacterial interaction. MAA introduced carboxylic groups for hydrogen bonding and improved selectivity. MMA added hydrophobicity and structural stability. VP improved solubility and flexibility. EGDMA cross-linked the polymer, forming *E. coli*-imprinted sites. AIBN initiated polymerization, creating a robust network with selective binding sites, critical for biosensor sensitivity.

The solution underwent 2 min of ultrasonication to eliminate dissolved gases, and then it was pre-polymerized for 30 min in an air-circulated gravity convection oven (HerathermTM, Thermo Fisher Scientific, Dreieich, Germany) at 65 °C. A suspension of *E. coli* OP50 (10^9^ CFU/mL, 375 µL) was centrifuged at 3000× *g* for 5 min. The resulting pellet was resuspended in 1.5 mL of pre-polymer solution. The modified SS-MWs were placed in this solution, and the container was sealed. The solution was polymerized for 11 h at 65 °C, with the initial 30 min involving rotation of the tube using a rotator at a fixed speed of 18 rpm to achieve a uniform coating. The oven’s internal thermostat ensured a consistent temperature throughout the process. Post-polymerization, CIP-MWs were rinsed sequentially for 20 s with a methanol-acetic acid solution (*v*/*v*: 9/1), methanol, and DI water for the elution of entrapped bacterial templates and creating binding cavities. This resulted in a CIP coating thickness of 2.2 ± 0.4 μm. Additionally, non-imprinted polymer (NIP) MWs were prepared under identical conditions but without *E. coli* templates as control electrodes.

### 2.5. Microfluidic Sensor

**Figure 1:**
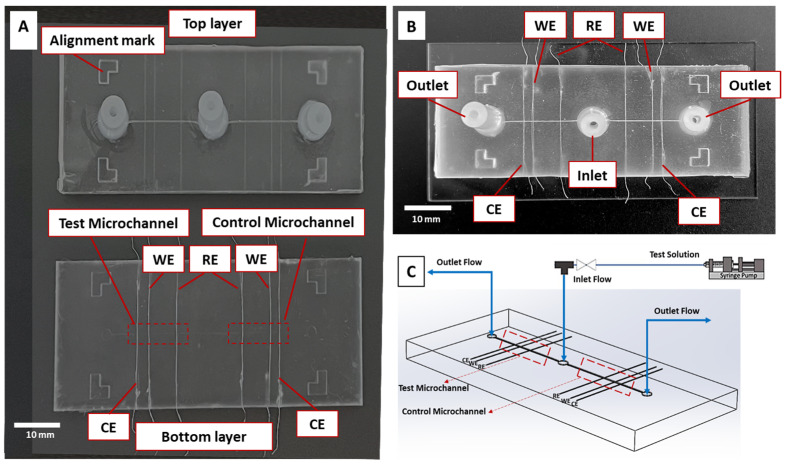
Impedimetric microfluidic bacteria sensor design and fabrication. (**A**) Upper and lower PDMS layers with integrated MWs. (**B**) Final microfluidic device post-plasma bonding of PDMS layers onto a glass slide. (**C**) Schematic of the sensor design illustrating flow directions and concurrent test and control measurement microchannels with CIP-MW and NIP-MW WEs, respectively. For REs and CEs, Ag-MWs and SS-MWs were used, respectively.

The microfluidic device was fabricated with two identical layers of polydimethylsiloxane (PDMS, Sylgard 184 silicone elastomer kit, Dow Corning Co., Midland, MI, USA), incorporating the sensor design. It featured two parallel microchannels (500 μm × 900 μm) with three perpendicular MW channels (130 μm ×130 μm) for integrating three electrodes essential for electrochemical measurements, along with inlet–outlet networks (Figure 1A). The thickness of each layer was 5 mm. CAD Solidworks 2023 software was used to design the master molds for PDMS replica molding, and 3D printing technology (Proto3000, Toronto, ON, Canada) was utilized to produce the master molds. The PDMS base was mixed with its curing agent at a 10:1 weight ratio and subsequently degassed for 15 min in a vacuum desiccator. The mixture was then poured onto the master molds and cured on a hot plate at 75 °C for 2 h. Following curing, the PDMS layers were removed, and MWs were incorporated into the lower layer channels. A CIP-MW served as the working electrode within the test microchannel, positioned in the central MW channel. Adjacent to the working electrode, an uncoated MW (SS-MW) was utilized as the counter electrode, maintained at a distance of 1.5 mm. Additionally, an MW with an Ag/AgCl coating functioned as the reference electrode and was positioned 3 mm away from the working electrode, to reduce interference with reactions at the working electrode. In the parallel microchannel, a similar arrangement was adopted, except for the working electrodes, where NIP-MWs were utilized. Oxygen plasma was employed to bond the two PDMS layers together, followed by bonding them to a glass slide (Figure 1B,C). Each sensor was fabricated and tested in five replicates.

### 2.6. Experimental Setup and Procedures

**Figure 2:**
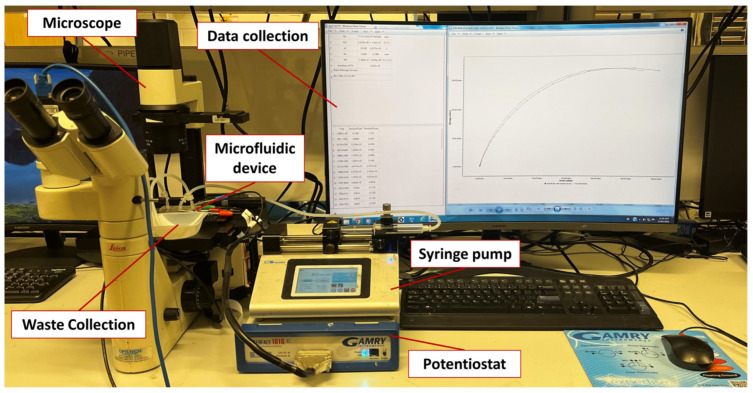
Experimental setup used to test the impedimetric microfluidic bacteria sensor.

Figure 2 demonstrates the experimental setup for impedimetric measurements. A DMIL LED standard inverted fluorescence microscope (Leica, Wetzlar, Germany) was employed to observe the microchannels and MWs throughout the measurements. EIS measurements were carried out with an Interface 1010E Potentiostat (Gamry Instruments, Warminster, PA, USA), with a standard three-electrode system, using a 0.1 M KCl solution containing 5 mM K_3_[Fe(CN)_6_] over a frequency range of 0.1–100 kHz, at room temperature, with a 10 mV perturbation amplitude. The sample was infused into the inlet microchannel at a 0.2 mL/min flow rate using a syringe pump (Legato 110, KD Scientific Inc., Holliston, MA, USA). Electrochemical spectra were fitted with an equivalent electrical circuit, and the charge transfer resistance (R_CT_) was measured pre- and post-bacteria exposure. Data acquisition and analysis were accomplished using Gamry Framework software Version 7.10.

The EIS measurement consisted of two stages where the charge transfer resistance of both device cells (microchannels with 3 electrodes) was measured before and after exposure to bacteria. In the initial stage, the electrolyte solution was infused through the microchannel, and measurements were taken. The resulting charge transfer resistance value (R_CT,1_) served as the baseline resistance. Following this, a bacteria suspension of specified count was introduced into the microchannels and allowed to incubate for 30 min to facilitate the bacteria binding to the CIP. The second EIS measurement (R_CT,2_) was conducted after flushing the microchannels with a blank electrolyte solution for 10 min to remove the unbonded bacteria from the device. The measurements were repeated for five replicate devices.

### 2.7. Analysis of Sensor Characterization Data

The change in the collected R_CT_ values was normalized by dividing the change in R_CT_ by the baseline R_CT_ of each experiment (before bacteria incubation) to standardize performance across devices [23]. The dose–response curve of the sensor was established using the normalized charge transfer resistance difference method according to Equation (1). Different bacterial counts were tested, and the resulting dose–response curve of ΔR/R_CT,1_ against the bacteria count was generated.

ΔR/R_CT,1_ = (R_CT,2_ − R_CT,1_)/R_CT,1_ (1)

Statistical analysis of the data was conducted using Minitab 16 software, with a significance level set at 0.05. The Mann–Whitney U test was employed to evaluate significant differences between pairs of means [24].

The sensor’s dynamic range was assessed by examining the statistical significance of normalized resistance responses between consecutive bacterial concentrations, with a significance level set at *p* < 0.05. To evaluate linearity, linear regression analysis was performed across the dose–response curve’s dynamic range, and the resulting R^2^ values were analyzed. The sensor’s sensitivity was determined from the slope of the linear portion of the response curve. The LOD and LOQ were defined as the bacteria counts corresponding to ΔR/R_CT,1_ values equivalent to the blank LB solution plus 3 and 10 times its standard deviation (SD), respectively [25].

## 3. Results and Discussion

### 3.1. EIS Analysis of the Microfluidic Device and Equivalent Electrical Circuit Fitting

**Figure 3:**
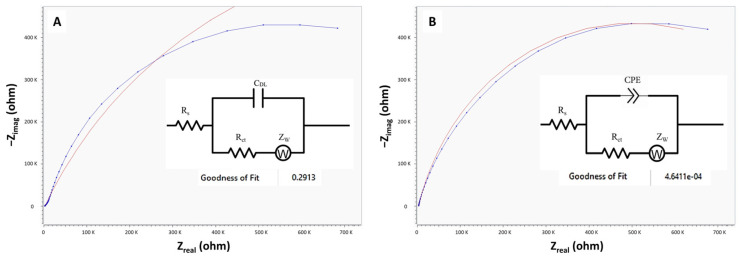
Electrochemical impedance spectroscopy (EIS) measurements and equivalent electrical circuits of the microfluidic sensor with CIP-MWs as the working electrode (WE) in the presence of K_3_[Fe(CN)_6_]/K_4_[Fe(CN)_6_] as the redox probe. (**A**) Standard Randles circuit diagram fit. (**B**) Modified Randles circuit diagram fit. Insets show the goodness of fit values. The blue lines represent the experimental data, while the red lines correspond to the fitted curves from the circuit models.

Electrochemical systems are often represented and analyzed through an equivalent circuit, which simulates the complex interplay of electrolyte/interface dynamics and redox reactions using electrical components such as resistors, capacitors, and sometimes inductors [26]. This approach enables a detailed investigation and evaluation of components within the system. By constructing and implementing these equivalent circuits, researchers gain insights into the underlying electrochemical processes, facilitating a deeper understanding of how these systems respond to changes in conditions like bacterial binding in microfluidic sensing platforms. In this section, we aimed to investigate the impedimetric characteristics of CIP-MWs upon binding with whole bacteria within our integrated microfluidic sensor. To explore this, we measured the Faradaic impedance of the sensor in the presence of the K_3_[Fe(CN)_6_]/K_4_[Fe(CN)_6_] redox couple as a probe as shown in Figure 2. The changes in the electrochemical properties of the redox probe were analyzed by subsequent fitting of this spectra to the standard Randles circuit, as depicted in Figure 3A. This equivalent circuit model helped explain the system’s behavior, incorporating the ohmic resistance of the electrolyte (R_s_), the Warburg impedance resulting from ion diffusion (Z_w_), the double layer capacitance (C_dl_), and the interfacial electron transfer resistance (R_CT_). However, fitting the resulting spectra with the standard Randles circuit yielded a poor goodness of fit with an error value of 0.29. This discrepancy was attributed to the presence of a constant phase element (CPE) in the system, which accounted for non-ideal behavior of a capacitor in a real system. To address this limitation, the model was refined by incorporating a modified Randles circuit with a CPE element, as shown in Figure 3B. This modification resulted in a significant improvement in the fitting quality with an acceptable goodness of fit and error value of 4.6 × 10^−4^, indicative of a better representation of the electrochemical processes occurring within the system. The observed enhancement underscores the importance of accounting for the non-ideal capacitive behavior inherent in the system’s response. These findings align with previous studies that highlight the necessity of CPE elements in impedance modeling of real systems as it defines nonhomogeneous charge distribution and surfaces in EIS experiments [27,28].

### 3.2. EIS Characterization of Bacteria Binding to CIP-MWs

**Figure 4:**
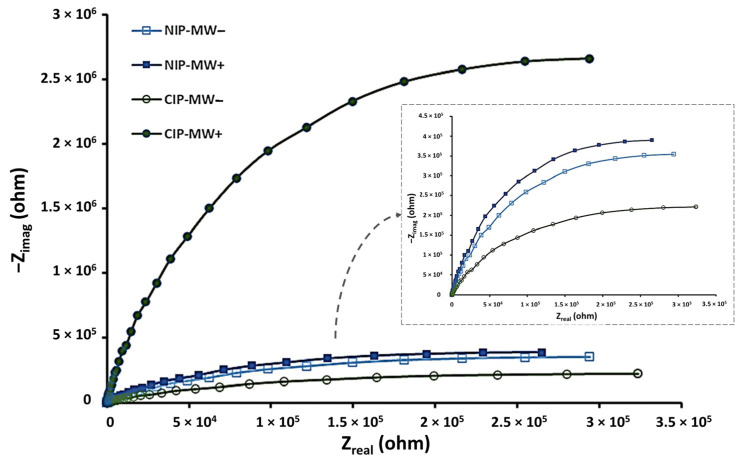
Electrochemical impedance spectroscopy (EIS) curves of microfluidic devices in 0.1 M KCl containing 5 mM K_3_[Fe(CN)_6_] with NIP-MWs and CIP-MWs serving as working electrodes. Minus and plus signs in the legend denote measurements obtained pre-and post-bacteria incubation, respectively. The inset shows an enlarged view of the NIP-MW (− and +) and CIP-MW data.

As bacteria bind to the surfaces of CIP-MWs, which serve as the WE in our sensor design, they may disrupt the charge transfer between the electrode surface and the electrolyte. This interaction can alter the interfacial properties of the WE. For EIS characterization of bacteria binding to CIP-MWs, parallel EIS measurements were conducted for microfluidic devices with NIP-MWs and CIP-MWs serving as WEs. Each test measurement was performed in two stages: before and after bacteria incubation (bacteria count 10^5^ CFU/mL). The resulting Nyquist plot spectra are depicted in Figure 4.

In a Nyquist plot, the semi-circle portion at high frequencies represents faradaic transfer of electrons at the surface of the WEs, while the low-frequency spectrum provides insights into the diffusion process of redox species between the electrolyte and the electrode surface. To understand the underlying mechanism of the observed response, the data were simulated using the modified Randles equivalent circuit model shown in Figure 3B. The R_s_ and Z_w_ impedance characterized the bulk properties and diffusion dynamics of the redox probe in the electrolyte solution, respectively, which remained unaffected by physicochemical transformations at the electrode surface [29] and, thus, were unaltered by CIP–bacteria binding. Conversely, parameters like R_CT_ and the capacitance of the double layer were contingent upon the dielectric and insulating characteristics at the electrode–electrolyte interface [30]. As mentioned earlier, a CPE was introduced into the circuit to better fit the impedance spectrum, reflecting defects and inhomogeneities of the layer [31]. Notably, R_CT_ was highly sensitive to electrode modifications resulting from *E. coli* bacteria binding to the CIP biorecognition layer. This binding process slowed interfacial electron transfer kinetics and elevated electron transfer resistance [32]. Consequently, the number of captured bacteria on the electrode surface could be inferred from the concentration-dependent electron transfer resistance of the redox probe, even at low analyte concentrations [29].

It can be seen from Figure 4 that the diameter of the semi-circle in Nyquist plot, indicating charge transfer resistance, was smaller for CIP-MWs than that for NIP-MWs. This difference may be due to the bacteria cavities and porosity in the CIPs, which facilitated ion transfer compared with the intact NIP coating. The semi-circle diameter for CIP-MWs after incubation with bacteria (CIP-MW+) increased significantly, directly indicating enhanced charge transfer resistance. This was likely due to the presence of captured bacteria by CIP-MWs, which hindered the transfer of redox ions between the electrode and the solution. For the control experiment performed in parallel with NIPs, there was a slight increase in the diameter of the semi-circle post-bacteria incubation (NIP-MW+). This could be due to small non-specific adsorption and bacterial attachment to NIP-MWs, which hindered ion transfer [33]. These results were consistent with prior studies, where the presence of bacteria was shown to significantly increase the charge transfer resistance due to hindrance in ion transfer pathways [34]. For instance, similar behavior has been observed in the work of Piskin et al. [35], where bacterial capture on bacteriophage-modified electrodes surfaces led to substantial R_CT_ changes. Our findings further demonstrate the robustness and superior sensitivity of CIPs compared with biological recognition agents like bacteriophages, particularly in comparison with NIP-MWs, highlighting the effectiveness of CIPs in creating selective cavities that facilitate targeted bacteria capture.

**Figure 5:**
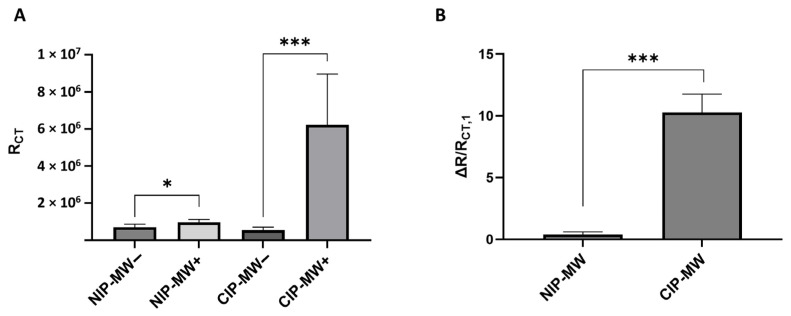
Charge transfer resistance (R_CT_) values for microfluidic devices in 0.1 M KCl containing 5 mM K_3_[Fe(CN)_6_] with NIP-MWs and CIP-MWs serving as working electrodes. (**A**) R_CT_ values obtained before normalization and (**B**) normalized R_CT_ change values. The minus and plus signs in the x axis indicate pre-and post-bacteria incubation measurements, respectively. The error bars are standard deviations (SD). *: *p*-value < 0.05; ***: *p*-value < 0.001.

Figure 5A presents the results for the measured R_CT_ values pre- and post-incubation with bacteria, for five replicates. While there was a slight increase in the R_CT_ values post-bacteria incubation for devices using NIP-MWs as WEs (*p*-value = 0.032), this increase in R_CT_ became a lot more significant for CIP-MWs (*p*-value = 0.0008). The normalized ΔR/R_CT,1_ result shown in Figure 5B reveals a 10-fold rise in the normalized charge transfer resistance change in CIP-MWs, confirming the bacteria cell attachment to selective cavities on CIP coatings, hindering redox ion transfer between the solution and the electrode surface. The *p*-values obtained from the Mann–Whitney U test demonstrated the statistical significance of the observed differences. Specifically, the significant increase in R_CT_ values for CIP-MWs (*p*-value = 0.0008) underscores the strong impact of bacteria capture on charge transfer resistance, in contrast to the more modest changes observed with NIP-MWs. This statistical analysis supports the conclusion that CIP-MWs offer a more effective and selective bacterial detection method, as evidenced by the highly significant difference in R_CT_ post-incubation.

### 3.3. Quantitative E. coli Bacteria Detection by EIS

**Figure 6:**
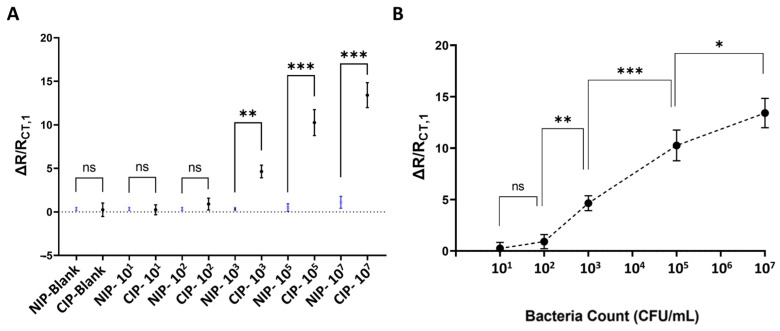
EIS-based microfluidic bacteria sensor characterization. (**A**) Normalized post-incubation charge transfer resistance shift of the microfluidic sensor with CIP-MWs and parallel control experiments utilizing NIP-MWs, when exposed to different bacteria counts. (**B**) The dose–response ΔR/R_CT,1_ curve established for the CIP-MW-based sensor. Error bars are standard deviations (SD). ns: non-significant; *: *p*-value < 0.05; **: *p*-value < 0.01; ***: *p*-value < 0.001.

The target bacteria detection in this study relied on measuring Faradaic impedance in the presence of the redox couple. It was hypothesized that the number of bacteria captured by CIP-MWs on the electrode surface would affect the transfer of redox ions between electrode and solution. Thus, the number of captured bacteria on the electrode could be determined by looking at how the redox probe’s electron transfer resistance changed, which could be measured using EIS. Following the examination of bacteria capturing properties exhibited by SS-MWs, NIP-MWs, and CIP-MWs and their impacts on the relevant concentration dependent parameter, R_CT_, the effect of bacteria counts on the sensor’s normalized change in charge transfer resistance was investigated. Using serial dilution, suspensions of *E. coli* bacteria with cell counts between 0 and 10^7^ CFU/mL were prepared. Figure 6A illustrates the normalized shift in charge transfer resistance of the sensor based on CIP-MW, alongside responses obtained from parallel control experiments utilizing NIP-MWs.

Figure 6A reveals that at bacterial counts ranging from 0 to 10^2^ CFU/mL, the response of the CIP sensor was not significantly different from the control experiments (*p*-value > 0.05). However, the difference became more significant by further increasing the bacteria count to 10^3^–10^7^ CFU/mL, demonstrating the dominant effect of the CIP coating as compared with NIP coatings with no significant responses. This behavior aligned with findings from previous works [19,36], where the sensitivity of CIP-based sensors significantly increased at higher bacterial concentrations, reinforcing the effectiveness of CIP-MWs in selective bacterial detection. The results also indicated that the detection capability of CIP-MWs at higher concentrations surpassed that of NIP-MWs, highlighting the advantages of using CIPs for enhanced sensitivity in pathogen detection applications.

Figure 6B illustrates the sensor’s dose–response curve generated from CIP-MWs across bacterial concentrations ranging from 0 to 10^7^ CFU/mL. The curve demonstrated negligible response at bacterial levels below 10^2^ CFI/mL, indicating the sensor’s lack was sensitivity at lower concentrations. A significant shift in charge transfer resistance was detected as bacterial concentrations increased from 10^2^ to 10^7^ CFU/mL. The statistical significance of the response peaked at bacterial counts of up to 10^5^ CFI/mL before declining, likely due to the saturation of the CIP cavities as the number of bacterial cells surpassed the binding capacity.

Statistical analysis indicated that the sensor’s dynamic range extended from 10^2^ to 10^7^ CFU/mL, based on the significant differences in consecutive readout signals within this interval. This range surpassed that of previous studies using CIP-MWs [19]. A linear regression analysis within this dynamic range yielded a goodness of fit (R^2^) value of 0.99, with a calculated sensitivity of 72.5 μS per CFU/mL, which is 10 times greater than the sensitivities reported in earlier research on conductometric *E. coli* detection using CIP-MWs [19].This broad linear detection range can facilitate the analysis of samples of unknown concentration via a simple dilution series, eliminating the need for sample concentration or pre-treatment steps. This versatility makes our approach particularly suitable for applications in clinical, food, and environmental industries. Furthermore, employing the 3-sigma and 10-sigma methods [37], the sensor’s LOD and LOQ were estimated to be 2 × 10^2^ CFU/mL and 1.4 × 10^4^ CFU/mL, respectively. These sensor characteristics were much lower than those obtained with the conductometric technique reported previously for microfluidic CIP-based bacteria sensors, representing a marked improvement in sensitivity.

To further enhance the detection limit of our sensor, several strategies can be employed. Optimizing the functionalization of CIPs by increasing their density on the microwires could improve the bacterial capture efficiency. Incorporating nanomaterials such as MXenes may boost the sensor’s electrical conductivity and sensitivity. Refining the EIS measurement conditions, including adjusting frequency ranges or signal amplitudes, could also enhance performance. Modifications to the microfluidic design, such as altering the channel dimensions and electrode surface area, might improve bacterial interaction. Additionally, further optimization of the CIP formulation could enhance binding and recognition capabilities.

Our established impedimetric microfluidic CIP-MW-based sensor achieves detection ranges and limits that are competitive with or superior to those of existing biosensors that use biological receptors for whole bacteria detection [38]. However, our proposed sensor has the advantage of the CIP’s durability and cost-efficiency over their biological counterparts [39]. Unlike biological recognition elements, such as aptamers, antibodies, and bacteriophages, which are often sensitive and lose functionality, CIPs maintain stability and performance. Additionally, the production of CIP-based films is controllable and facile, and the material is inexpensive, biocompatible, and biodegradable. The developed sensor’s performance surpasses that of other CIP-based bacterial sensing methods such as frequency-based techniques using quartz crystal microbalance (QCM) [40,41]. Furthermore, impedimetric sensing offers several added advantages over QCM techniques. Unlike QCM, which relies on precise frequency measurements and can be complex and costly, impedimetric sensing simplifies the detection process by directly measuring changes in electrical impedance. This approach is particularly beneficial for detecting low concentrations of analytes, where QCM’s frequency shifts can become less accurate and more difficult to measure. Additionally, impedimetric sensors are often more robust, less sensitive to environmental variations such as temperature and viscosity changes, and generally more adaptable for a wide range of applications. These factors make impedimetric sensing a more versatile and cost-effective alternative for various biosensing needs [41]. This validates the suitability of integrating an EIS-based microfluidic biosensing strategy with CIPs for the creation of pathogen sensors with high sensitivity, presenting a genuine alternative to conventional methods. Bringing together the advantages of biosensors and CIPs, this method is promising for pathogen detection, offering the advantages of enhanced sensitivity, selectivity, simplicity, low cost, and stability.

## 4. Conclusions

A low-cost, miniaturized, and label-free microfluidic biosensor for *E. coli* bacteria detection based on CIPs was designed and fabricated. The sensing capacity of the resulting CIP-based biosensor toward the target bacteria was assessed by the EIS characterization technique. The developed biosensor, employing CIP-MWs as working electrodes in PDMS microchannels, demonstrated enhanced sensitivity to bacterial presence. Using EIS measurements, an increase in the charge transfer resistance of CIP-MWs after exposure to bacteria was detected, enabling quantification between 10^2^ and 10^7^ CFU/mL with detection and quantification limits of 2 × 10^2^ CFU/mL and 1.4 × 10^4^ CFU/mL, respectively. The wide linear detection range of the reported biosensor can enable the analysis of real samples without pre-treatment or concentration steps. With its potential for cost-effectiveness, durability, portability, and real-time monitoring, the developed sensor presents a promising solution for waterborne pathogen detection at the PoN.

## Data Availability

The original contributions presented in the study are included in the article material; further inquiries can be directed to the corresponding author/s.
